# Quantitative Flow Ratio Is Related to Intraluminal Coronary Stenosis Parameters as Assessed with Optical Coherence Tomography

**DOI:** 10.3390/jcm10091856

**Published:** 2021-04-24

**Authors:** Andrea Milzi, Rosalia Dettori, Kathrin Burgmaier, Nikolaus Marx, Sebastian Reith, Mathias Burgmaier

**Affiliations:** 1 Department of Cardiology, University Hospital of the RWTH Aachen, D-52074 Aachen, Germany; rdettori@ukaachen.de (R.D.); nmarx@ukaachen.de (N.M.); sreith@ukaachen.de (S.R.); mburgmaier@ukaachen.de (M.B.); 2 Department of Pediatrics, University Hospital Cologne, D-50441 Cologne, Germany; kathrin.burgmaier@uk-koeln.de

**Keywords:** optical coherence tomography, quantitative flow ratio, coronary artery disease, coronary physiology, plaque vulnerability

## Abstract

**Background:** Quantitative flow ratio (QFR) is a novel method for assessing hemodynamic relevance of a coronary lesion based on angiographic projections without the need of a pressure wire. Various studies demonstrated that QFR consistently related to fractional flow reserve (FFR); however, it is still unclear to what extent QFR reflects intraluminal stenosis parameters. Given that optical coherence tomography (OCT) is currently the gold standard to assess intraluminal stenosis parameters, we investigated the relationship between OCT-derived lesion geometry and QFR. **Methods:** We determined QFR in 97 lesions from 87 patients who underwent coronary angiography and OCT due to stable angina. QFR was measured with proprietary software and compared with OCT-based assessment of intraluminal stenosis parameters as well as lesion morphology. **Results:** Mean QFR was 0.79 ± 0.10. QFR demonstrated a consistent association with FFR (*r* = 0.834, *p* < 0.001). Interestingly, QFR was associated with OCT-derived parameters such as minimal lumen area (MLA, *r* = 0.390, *p* = 0.015), percent area stenosis (*r* = 0.412, *p* < 0.001), minimal lumen diameter (MLD, *r* = 0.395, *p* < 0.001), and percent diameter stenosis (*r* = 0.400, *p* < 0.001). Both minimal luminal area (ROC = 0.734, optimal cut-off 1.75 mm^2^) and minimal luminal diameter (ROC = 0.714, optimal cut-off 1.59 mm) presented a good diagnostic accuracy in diagnosing hemodynamic relevance (QFR ≤ 0.80). There was no significant association between QFR and anatomic features of plaque vulnerability. **Conclusion:** OCT-derived intraluminal stenosis parameters are related to QFR values and predict hemodynamic lesion relevance. The data supports the validity of QFR as 3D-vessel reconstruction method to assess coronary physiology without the need of a pressure wire.

## 1. Background

Chronic coronary syndromes (CCS) are stable manifestations of coronary artery disease (CAD). In CCS, revascularization is indicated with proof of ischemia [1,2]. This may be obtained by means of non-invasive testing, such as stress echocardiography, single-photon emission computerized tomography or cardiac magnetic resonance. On the other hand, invasive coronary angiography with functional testing using a pressure wire remains the gold standard to assess a lesion’s hemodynamic relevance [2]. Different wire based types of functional tests are currently employed such as fractional flow reserve (FFR), instantaneous wave-free ratio (iFR), or resting full-cycle ratio (RFR); however, these types of tests inherently bear a relevant risk of coronary vessel damage due to the necessity of wire advancement into the coronary artery and past the coronary stenosis, which may potentially cause vascular complications as dissections or plaque activation. The most widely employed wire-based method is FFR, which requires the application of intracoronary or intravenous drugs such as adenosine, which may occasionally arouse arrhythmias or bronchospasm. Overall, major complications are reported to occur in 0.1% to 1.5% of the FFR measurements [3,4]. Therefore, in order to minimize the risk of complications, an interesting option to assess hemodynamic relevance of coronary lesions is quantitative flow ratio (QFR), a novel intracoronary wire-free technique able to assess the pressure drop in the vessel based on a three-dimensional reconstruction of the atherosclerotic vessel and on the flow velocity of contrast medium. QFR has already been tested in CCS as a reliable, safe, and cost-effective alternative to FFR to assess functional significance of coronary stenosis [5,6,7,8]. Based on these studies, a QFR-value ≤ 0.80 has been defined as marker of the functional significance of a coronary stenosis [5,6,7,8]. Furthermore, a relevant diagnostic agreement of QFR has been proven also for iFR [9].

However, as QFR is dependent on parameters such as flow velocity of contrast medium and this may be compromised in conditions such as microvascular dysfunction, it is unclear to what extent QFR values actually reflect intraluminal stenosis parameters. Given that optical coherence tomography (OCT) is currently the gold standard to assess intraluminal stenosis parameters [10,11], we investigated the relationship between OCT-derived lesion geometry and QFR; in addition, the association of QFR values with plaque vulnerability is still largely unexplored and is also investigated in this study.

## 2. Materials and Methods

### 2.1. Patient Selection

This study was a post-hoc analysis of our institutional OCT registry, on which some previous studies were based [12,13,14,15,16,17,18]. Written informed consent of all patients for inclusion in the registry was obtained previous to coronary angiography and OCT. Exclusion criteria were left main coronary artery stenosis, bifurcation and bypass graft lesions, ongoing acute coronary syndrome, an acute or chronic renal insufficiency, and pregnancy. Tandem lesions, defined as two or more lesions separated by a normal tract of coronary vessel, were not included in the analysis. On the contrary, long diffuse lesions (i.e., lesions with a length of >20 mm) were not excluded. The registry was approved by the local ethics committee and is in accordance with the declaration of Helsinki on ethical principles for medical research involving human subjects.

**Figure 1:**
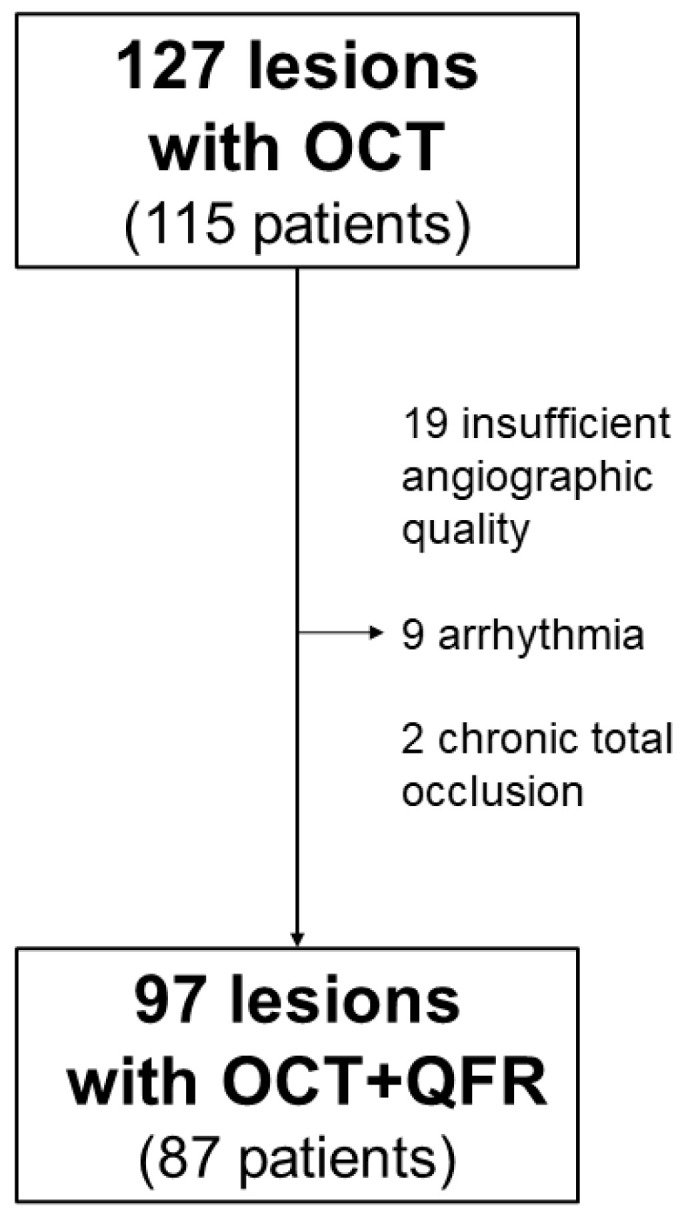
Flowchart depicting study inclusion.

In the initially included 115 patients who underwent coronary angiography and OCT due to CCS at the Department of Cardiology of the University Hospital of the RWTH Aachen, 127 lesions were initially screened for inclusion. Of these, 30 were excluded for reasons impeding QFR analysis (19 for insufficient angiographic image quality, 9 for arrhythmia, 2 for chronic total occlusions). This resulted in 97 lesions suitable for both QFR and OCT-evaluation in 87 patients. A study inclusion flowchart is depicted in Figure 1.

### 2.2. QFR-Analysis

**Figure 2:**
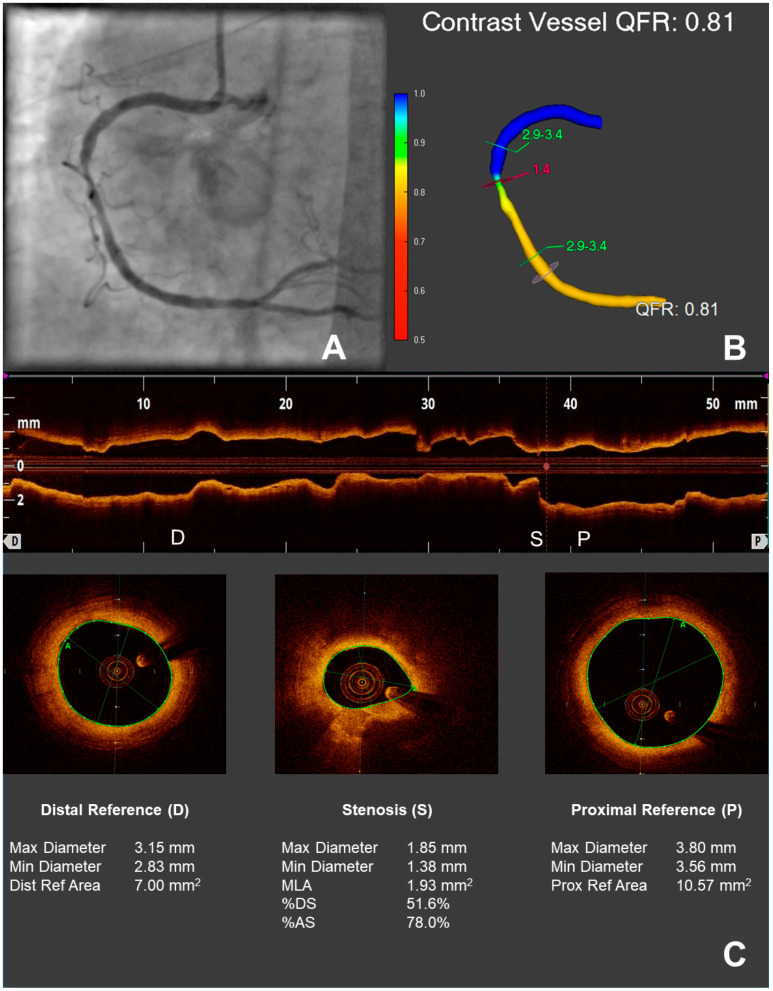
Assessment of coronary physiology with QFR and of stenosis geometry with OCT. A stenosis of the right coronary artery is depicted in (**A**). In (**B**), the derived 3D vessel reconstruction with the calculated QFR is shown. In (**C**), assessment of the geometry of the same stenosis through OCT: in the upper panel, longitudinal view with markers for proximal reference (P), maximal stenosis (S), and distal reference (D); in the lower panels, cross sections for proximal reference, maximal stenosis, and distal reference with the respective intraluminal measurements.

QFR analysis was performed using Medis proprietary software (QAngio suite and QFR, Medis, Leiden, The Netherlands) and analogously to previous studies [5,6,7,8]. In short, two angiographic images with a difference >25° and minimal overlapping of the target lesion were selected. In previous prospective studies [5,6,7,8], optimized protocols for image acquisition in order to maximize image quality for QFR analysis were employed; due to the retrospective nature of this work, such protocols could not be applied. Image quality was assessed by the operator prior to analysis: if the quality did not allow adequate QFR analysis (for instance, due to excessive vessel overlap or insufficient vessel filling), the lesion was excluded from analysis. Standard acquisition of angiographic images at the host institution was performed with at least 10 frames/second. In all included vessels, image quality was at least sufficient for adequate QFR analysis. Nitroglycerine had been applied previous to image acquisition according to standard operating procedures. Following image choice, end-diastolic frames were selected, the target segment of the considered vessel was manually determined and the automatically detected vessel contour was checked and, when necessary, corrected. Flow QFR was obtained by semiquantitative assessment of contrast dye flow, as allowed by the commercial software used. Flow QFR was used for further analysis. An example of the three-dimensional reconstruction of a vessel including flow-QFR calculation is depicted in Figure 2, aside to angiographic view of the vessel.

MLD, percent diameter stenosis and stenosis length based on the simulation were registered.

QFR analysis was performed offline and retrospectively by certified QFR-software user (AM), blinded to results of OCT and FFR analysis as well as to clinical features of patients.

### 2.3. OCT-Image Acquisition and Analysis

OCT image acquisition and analysis was performed as described elsewhere [15,16,19]. An example of OCT-based analysis of stenosis geometry is depicted in Figure 2C.

Analysis of plaque morphology was only possible on native coronary lesions (*n* = 80). Minimal and mean fibrous cap thickness (FCT) were determined solely on lipid plaques, as per definition [19].

OCT analysis was performed offline and retrospectively by an experienced observer (AM or SR) blinded to both QFR and FFR results as well as to clinical features of patients.

### 2.4. Statistical Analysis

Clinical features and laboratory values were analyzed on a per-patient basis, lesion characteristics on a per-lesion basis. Continuous variables were reported as mean ± standard deviation, dichotomic variables as count (percentage). Association of FFR with QFR, as well as of OCT-derived intraluminal stenosis parameters with QFR was assessed by means of linear and non-linear regression. Distribution of continuous variables depending on QFR ≤ 0.80 or not was assessed by *t*-test, distribution of dichotomic ones by Pearson’s chi-squared test. In order to assess diagnostic efficiency of intraluminal stenosis parameters in predicting a QFR ≤ 0.80, we performed ROC analysis; diagnostic efficiency was classified as previously described [20]. The value with the highest Youden index was defined as optimal cut off.

All analyses were performed using SPSS statistical package (v. 26.0, IBM Corp., Armonk, NY, USA). Statistical significance was awarded for *p* < 0.05.

## 3. Results

### 3.1. Patient and Lesion Characteristics

Relevant clinical characteristics of the 87 included patients are reported in Table 1. The 97 vessels included in the study presented an average OCT-based MLD of 1.3 ± 0.3 mm, an average OCT-based MLA of 1.9 ± 0.9 mm^2^ and an average QFR of 0.79 ± 0.10. Further data are reported in Table 2.

### 3.2. Association between FFR and QFR

**Figure 3:**
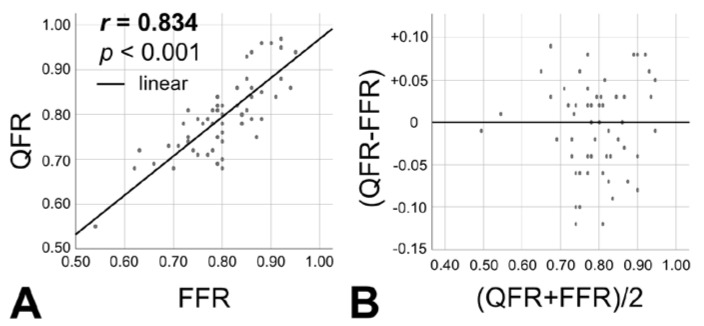
**Association between FFR and QFR.** A strong association between the two modalities to assess coronary physiology is present; in (**A**), linear regression is presented; in (**B**), Bland - Altman plot is shown.

In the subgroup of lesions (*n* = 60), which underwent assessment of the hemodynamic relevance with FFR, we could show a significant concordance between FFR and QFR values (*r* = 0.834, *p* < 0.001), as shown in Figure 3.

### 3.3. Association between OCT and Quantitative Angiography-Derived Stenosis Parameters

**Figure 4:**
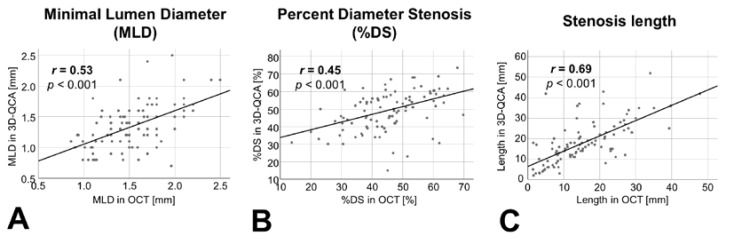
**Association between OCT and 3D-quantitative coronary angiography (3D-QCA) in assessing stenosis geometry.** A strong linear association between the two modalities to assess stenosis parameters could be demonstrated; exemplary, minimal lumen diameter (MLD, in (**A**)), percent diameter stenosis (%DS, in (**B**) and stenosis length (in (**C**)) are presented.

In order to assess the robustness of the quantitative angiography-based 3D vessel reconstruction used for QFR computation, we compared stenosis parameters obtained through quantitative angiography with those derived from OCT analysis. A linear association between different geometric stenosis parameters (minimal lumen diameter, percent diameter stenosis, stenosis length) assessed in the two modalities could be demonstrated, as shown in Figure 4.

### 3.4. Association between OCT-Derived Stenosis Parameters and QFR

**Figure 5:**
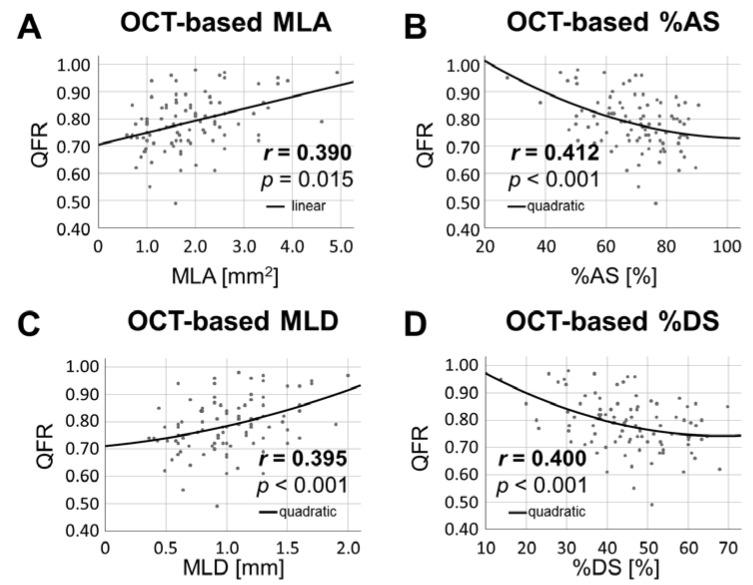
**Association between OCT-derived stenosis parameters and QFR.** Physiological assessment of coronary lesions through QFR was consistently associated with OCT-derived stenosis parameters as MLA (**A**), percent area stenosis (**B**), MLD (**C**) and percent diameter stenosis (**D**). Abbreviations: MLA: minimal lumen area, %AS = percent area stenosis, MLD: minimal lumen diameter, %DS = percent diameter stenosis.

More interestingly, OCT-derived parameters such as minimal lumen area (*r* = 0.390, *p* = 0.015), percent area stenosis (*r* = 0.412, *p* < 0.001), minimal lumen diameter (*r* = 0.395, *p* < 0.001) and percent diameter stenosis (*r* = 0.400, *p* < 0.001) were consistently associated with QFR, as depicted in Figure 5.

**Figure 6:**
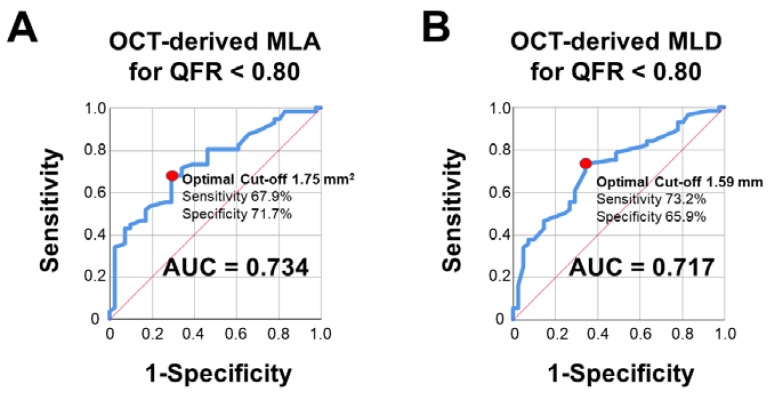
**Diagnostic efficiency of OCT-derived stenosis parameters in predicting pathological QFR (≤0.80).** Both MLA (**A**) and MLD (**B**) presented a good diagnostic efficiency in detecting hemodynamically relevant stenoses according to QFR. Abbreviations as in previous figures.

After we demonstrated a consistent association of QFR with OCT-derived intraluminal stenosis parameters, we aimed to assess the diagnostic efficiency of MLA and MLD in predicting QFR ≤ 0.80. Both OCT-derived MLA (AUC = 0.734, optimal cut-off 1.75 mm^2^, sensitivity 67.9%, specificity 71.7%) and MLD (AUC = 0.717, optimal cut-off 1.59 mm, sensitivity 73.2%, specificity 65.9%) presented a good diagnostic accuracy in diagnosing a QFR ≤ 0.80. For further details please refer to Figure 6.

### 3.5. Association between Plaque Vulnerability and QFR

Given the significant relationship between lesion geometry and QFR, we next tested the association of measures of plaque vulnerability as assessed by OCT with QFR in native coronary lesions. We could not find any statistically significant difference between patients with pathological (≤0.80) and normal (>0.80) QFR regarding mean FCT (128 ± 28 vs. 144 ± 39 µm, *p* = 0.152), minimal FCT (86 ± 32 vs. 94 ± 32 µm, *p* = 0.497) or presence of macrophage infiltration (48.9% vs. 50%, *p* = 0.925), suggesting no association between plaque vulnerability and QFR.

## 4. Discussion

The main findings of our study are:

1. Stenosis parameters measured by OCT are significantly associated with QFR.
2. Furthermore, stenosis parameters in OCT are consistent with assessment of lesion morphology by 3D-quantitative coronary angiography.
3. An OCT-derived MLA of 1.75 mm^2^ and an MLD of 1.59 mm were the optimal cut-offs for predicting hemodynamic relevance of coronary lesions according to QFR.
4. Plaque vulnerability is not associated to QFR.

QFR is a novel, wire-free technique assessing hemodynamic relevance of coronary stenoses, based only on two angiographic projections. Although a good association of QFR with FFR and iFR has been shown in previous studies [5,6,7,8,9], still no study addressed the association of QFR measurements with stenosis geometry, as assessed with intravascular imaging and specifically OCT. This is a relevant point, particularly considering that QFR differently from wire-based techniques is based on a 3D-reconstruction of the diseased vessels.

### 4.1. QFR Is Associated with OCT-Derived Intraluminal Stenosis Parameter

First of all, we found an excellent association between QFR and FFR measurements in a subgroup of 60 patients. This is consistent with previous studies [5,6,7,8] and, in analogy, with studies comparing QFR to other methods able to assess coronary physiology, such as iFR [9]. This finding confirms the robustness of our analysis. Furthermore, the 3D-reconstruction of the vessel used from the commercial software for computing QFR effectively depicts stenosis geometry, as shown by the linear associations between stenosis parameters assessed by quantitative angiography and OCT.

More interestingly, we could show a strong association of OCT-derived intraluminal stenosis parameter, as MLA, MLD and percent area stenosis with QFR. This parallels the finding of analogue studies carried out comparing FFR with the assessment of lesion geometry by intravascular imaging and particularly with OCT [16,21,22,23,24]. This suggests that functional assessment of a coronary lesion by QFR, as well as by its wire-based counterparts, presents a strict association with the anatomic severity of lesions. Furthermore, the optimal cut-offs for MLA (1.75 mm^2^) and MLD (1.59 mm) derived from our analysis are similar to those found in previous studies comparing FFR and OCT [16,21,22,23,24]. This, again, highlights the robustness of QFR, which seems to share the same relationship with anatomic severity already detected for FFR, the current gold standard for assessing coronary flow. It has to be remarked, though, that the overlap between stenosis geometry and physiological assessment may not be exact. This is due to the many factors determining flow, which are not limited to geometrical properties of the vessel but also include, for instance, perfusion pressure, vascular resistance, microvascular function, and neurohumoral factors.

### 4.2. QFR Is not Associated with Plaque Vulnerability

A recent study by Kanno et al. [25] showed that a lower QFR may be associated to a higher rate of thin capped fibroatheromas in the investigated lesions. We aimed to extend the current knowledge by further assessing plaque vulnerability, as determined by OCT, in dependence of QFR. Surprisingly, we found that minimal and mean FCT as well as the rate of macrophage infiltration, as the most established parameters of plaque vulnerability, do not differ in lesions with or without hemodynamic relevance according to QFR. In the light of these data, it might be tempting to speculate that QFR, in spite of its excellent ability to assess coronary physiology, cannot provide significant information regarding plaque vulnerability. A recent post-hoc analysis of the PROSPECT and IBIS-4 study pointed in this direction, showing that in non flow-limiting lesions QFR may be useful in predicting major cardiovascular events in addition to coronary plaque morphology [26]. To this extent, even a QFR over the accepted threshold for the definition of hemodynamic relevance (i.e., >0.80) seems to yield a potential prognostic value. Interestingly, the fact that this prognostic value is additional to the presence of high-risk plaque morphological feature may be due to the ability of QFR to evaluate different features (such as microvascular dysfunction [27]), which may nevertheless be relevant for prognosis. On the other hand, OCT is currently the gold standard in the assessment of plaque vulnerability [10,11,19], which has a direct impact on cardiac events [28], if not timely sealed through coronary intervention [14]. Furthermore, the role of ischemia testing alone has been questioned by the results of the recent ISCHEMIA trial, where no evidence of the superiority of an initial invasive strategy compared to a conservative one regarding myocardial infarction and all-cause death could be shown in patients with CCS and proof of ischemia [29]. Although the results of this trial were largely dependent from a higher incidence of periprocedural myocardial infarction in patients in the invasive strategy group, and in the long turn patients in the conservative group showed a trend to more frequent coronary events and need for revascularization, these results question a purely ischemia-based strategy. A hybrid approach, taking into account not only hemodynamic relevance of a stenosis, but also plaque vulnerability and patient characteristics, may yield relevant improvements in patient prognosis. Therefore, these two modalities (OCT and QFR) might complement each other in a modern, multimodal assessment of coronary lesions, as previously suggested also for wire-based methods—but, in the case of QFR, with the advantage of lower risks for patients.

## 5. Limitations

Although being, to the best of our knowledge, the first study exploring the association of OCT-derived intraluminal stenosis parameter with QFR, our study population is still relatively small; therefore, our findings need to be confirmed in larger study cohorts. This is particularly true for the cut-off values that we found. Furthermore, the exclusion of severely calcified, tortuous, or tandem lesions impeding the safe passage of the OCT catheter might have reduced the informative value of our study regarding very complex coronary lesions. Due to the study design, we further excluded patients with relevant left main disease; therefore, we are unable to draw any conclusion regarding this specific subpopulation.

## 6. Conclusions

QFR and OCT-derived intraluminal measurements are significantly associated. Our data support the validity of QFR. On the other hand, QFR does not show any association with plaque vulnerability, suggesting that this novel technique might be effectively complemented by intravascular imaging in order to obtain a modern, multimodal assessment of coronary lesions.

## Figures and Tables

**Table 1:** Study population characteristics.

	*n* = 87
Age (yrs)	65.4 ± 11.6
Male sex (*n*, %)	62 (71.3)
LVEF (%)	52.9 ± 9.2
**CV risk profile**	
Diabetes (*n*,%)	28 (32.2)
HbA1c (%)	6.2 ± 1.4
BMI (kg/m^2^)	29.8 ± 5.3
Hypertension (*n*,%)	66 (75.9)
Family history of CAD (*n*,%)	33 (37.9)
Nicotin use (*n*,%)	24 (27.6)
Packyears (PY)	18.5 ± 28.4
Hyperlipidemia (*n*, %)	56 (64.4)
Cholesterol (mg/dL)	187 ± 53
LDLc (mg/dL)	118 ± 42
HDLc (mg/dL)	47 ± 14
Triglycerides (mg/dL)	147 ± 103
CRP (mg/L)	7 ± 8
**Medication**	
Aspirin (*n*,%)	77 (88)
Statin (*n*,%)	73 (84)
Beta Blocker (*n*,%)	63 (72)
ACEi or ARB (*n*,%)	65 (75)

Abbreviations: LVEF = left ventricular ejection fraction; BMI = body mass index; CAD = coronary artery disease; ACEi = angiotensin converting enzyme inhibitor; ARB = angiotensin receptor blocker.

**Table 2:** Lesion characteristics.

	*n* = 97
Native vessel disease (*n*,%)	80 (82.5)
In-Stent Restenosis (*n*,%)	17 (17.5)
**Vessel**	
LAD (*n*,%)	59 (60.8)
LCx (*n*,%)	13 (13.4)
RCA (*n*,%)	18 (18.6)
Diagonal Branch (*n*,%)	1 (1)
Obtuse branch (*n*,%)	5 (5.2)
RIM (*n*,%)	1 (1)
**QCA/QFR based characteristics**	
QFR	0.79 ± 0.10
QFR ≤ 0.80	56 (57.7)
MLD in QCA (mm)	1.3 ± 0.3
%DS in QCA (%)	48.6 ± 11.2
**OCT based characteristics**	
Proximal RD (mm)	3.0 ± 0.5
Proximal Reference Area (mm^2^)	7.1 ± 2.5
Distal RD (mm)	2.7 ± 0.6
Distal Reference Area (mm^2^)	5.9 ± 2.8
MLD in OCT (mm)	1.3 ± 0.3
MLA in OCT (mm^2^)	1.9 ± 0.9
Stenosis eccentricity (%)	25.1 ± 11.9
%DS in OCT (%)	45.3 ± 11.4
%AS in OCT (%)	69.5 ± 12.5
Mean FCT (µm)	135 ± 34
Minimal FCT (µm)	89 ± 33
Macrophage infiltration (*n*,%)	40 (41.2%)

Abbreviations: LAD = left anterior descending; LCx = left circumflex; RCA = right coronary artery; RIM = Ramus intermedius; QCA = quantitative coronary angiography; QF*r* = quantitative flow ratio; OCT = optical coherence tomography; MLA = minimal lumen area; MLD = minimal lumen diameter; RD = reference diameter; %DS = percent diameter stenosis; %AS = percent area stenosis; FCT = fibrous cap thickness.

## Data Availability

The data presented in this study are available on request from the corresponding author. The data are not publicly available due to privacy issues.
